# Effect of *Kaempferia parviflora* Supplementation in Semen Extenders on Post-Thaw Sperm Quality in Thai Native Bulls

**DOI:** 10.3390/ani15070962

**Published:** 2025-03-27

**Authors:** Thanapon Loetjettanarom, Supakorn Authaida, Wuttigrai Boonkum, Vibuntita Chankitisakul

**Affiliations:** 1 Department of Animal Science, Faculty of Agriculture, Khon Kaen University, Khon Kaen 40002, Thailand; thanaponlo@kkumail.com (T.L.); supakorn.u@kkumail.com (S.A.); wuttbo@kku.ac.th (W.B.); 2 The Research and Development Network Center of Animal Breeding and Omics, Khon Kaen University, Khon Kaen 40002, Thailand

**Keywords:** freezing semen, antioxidant, lipid peroxidation, cattle

## Abstract

Cryopreservation is a widely used technique for long-term semen storage; however, it can induce oxidative stress, compromising sperm quality. This study examines the effect of *Kaempferia parviflora* (KP) supplementation in semen extenders on the post-thaw sperm quality of Thai native bulls. Fresh semen was collected and evaluated for motility, viability, and concentration prior to cryopreservation. Semen samples were allocated to four treatment groups: 0 mg/mL (control), 0.5 mg/mL, 1 mg/mL, and 1.5 mg/mL KP. Subsequently, the samples were frozen, then thawed, and analyzed for sperm motility, viability, membrane integrity, and oxidative stress. The results showed that supplementation with 1.0 mg/mL KP significantly improved sperm motility (46.29 ± 2.66%) and viability (43.42 ± 2.15%) compared to the control (40.77 ± 2.76% and 38.63 ± 2.66%, respectively). Membrane integrity was also enhanced (47.64 ± 1.18% vs. 41.85 ± 1.98% in the control), while oxidative stress levels were reduced (MDA concentration: 2.33 ± 0.29 µM/mL vs. 2.73 ± 0.33 µM/mL in the control). However, the highest concentration (1.5 mg/mL KP) negatively affected sperm quality, with reduced motility (36.97 ± 3.32%), viability (30.88 ± 3.02%), and membrane integrity (35.64 ± 1.61%). These findings suggest that 1 mg/mL KP is the optimal concentration for improving post-thaw semen quality in cryopreservation protocols for Thai native bulls.

## Simple Summary

Freezing semen can cause damage due to oxidative stress, which affects sperm quality. To help protect the sperm, antioxidants can be added to the freezing extender. This study tested whether *Kaempferia parviflora* (KP), a natural antioxidant, could improve the quality of frozen-thawed sperm from Thai native bulls. Semen samples were treated with different amounts of KP (0, 0.5, 1.0, and 1.5 mg/mL) before freezing. After thawing, the sperm were examined for motility, viability, membrane integrity, and oxidative stress levels. The results showed that adding 1.0 mg/mL of KP significantly improved sperm quality while reducing oxidative stress. However, a higher dose (1.5 mg/mL) had negative effects. These findings suggest that 1.0 mg/mL of KP is the best amount to use for improving sperm quality after freezing, which could be beneficial for cattle breeding programs.

## 1. Introduction

Cryopreservation has become an essential tool in livestock breeding programs, enabling the storage and transport of genetic material across different regions and generations. This technology is crucial for preserving genetic diversity, improving breeding efficiency, and supporting conservation efforts, particularly for indigenous breeds that play a critical role in local agricultural systems. One such breed is the Thai native bull (*Bos indicus*), which has been integral to Thailand’s agricultural landscape for centuries. These bulls are particularly known for their adaptability to tropical climates, disease resistance, and ability to thrive in environments with limited forage quality [1].

Despite their importance, Thai native bull populations have been declining due to factors such as shifts in agricultural practices, economic changes [2], and the increasing popularity of crossbreeding programs focused on increasing beef production by replacing local breeds with exotic cattle [3]. This decline poses a threat to the genetic diversity of native breeds, highlighting the need for conservation strategies like semen cryopreservation.

Semen cryopreservation provides a promising method for preserving the genetic material of Thai native bulls, enabling breeders to safeguard the unique traits of the breed for future generations. To overcome the geographic and reproductive limitations associated with live bulls, cryopreserved semen can be stored indefinitely in liquid nitrogen and used in artificial insemination (AI) programs [4]. Moreover, the ability to distribute semen across regions facilitates genetic exchange and reduces the risks associated with live animal transport, such as disease transmission and stress [5].

However, cryopreservation, particularly the freezing and thawing stages, often results in significant sperm cell damage, including reduced motility, viability, and fertility [6]. This damage is primarily caused by oxidative stress, which arises when excess reactive oxygen species (ROS) are produced during cryopreservation. ROS can induce lipid peroxidation, protein denaturation, and DNA fragmentation, ultimately impairing sperm function and lowering AI success rates [7]. Thus, developing effective semen extenders enriched with cryoprotectants and antioxidants is critical for minimizing cryodamage and improving post-thaw sperm quality.

In recent developments, natural antioxidants have gained attention as potential additives to semen extenders for bull cryopreservation, enhancing sperm motility and viability due to their ROS-scavenging properties and ability to mitigate oxidative stress [8,9,10]. Additionally, there is growing interest in the potential side effects and cost-effectiveness of plant-derived antioxidants, which are considered safer and more sustainable alternatives [11].

*Kaempferia parviflora* (KP), also known as Thai ginger, is a medicinal plant native to Southeast Asia that is valued for its health-promoting properties. Rhizome extracts from KP have demonstrated strong antioxidant activity, effectively scavenging free radicals and inhibiting lipid peroxidation by 80% at a 20 μg/mL concentration, indicating a substantial antioxidative capacity [12]. The efficacy of KP in mitigating oxidative stress during cryopreservation has been previously demonstrated in rooster sperm following oral administration [13]. However, this approach is limited by practical challenges associated with KP’s palatability related to its taste and odor, which restricts its practical application. To address this, our study explores an alternative strategy: the direct supplementation of KP in the semen extender. This novel approach aims to harness KP’s antioxidant properties more effectively, offering a promising yet unexplored method for enhancing post-thaw semen quality.

This study aims to assess the effects of KP supplementation at different concentrations (0.5 mg/mL, 1 mg/mL, and 1.5 mg/mL) on the post-thaw semen quality of Thai native bulls. Key parameters, including sperm motility, viability, membrane integrity, and oxidative stress, were assessed to determine the optimal KP concentration for improving semen quality after cryopreservation.

## 2. Materials and Methods

### 2.1. KP Extract

KP extract powder was purchased from AsianBioplex Co. (Bangphra, Si-Racha, Chonburi, Thailand). The bulk density, moisture content, and flavonoid content of the powder were 0.30–0.60 g/cc, <10%, and >1.5%, respectively. Deionized water was used as the extraction solvent.

### 2.2. Determination of Total Phenolic Content (TPC)

The TPC is a widely recognized indicator of the antioxidant capacity of plant extracts. In this study, the TPC was determined using the Folin-Ciocalteu (F-C) method, in accordance with a procedure adapted from the study by Adebiyi et al. [14]. Gallic acid was used as the standard reference compound. Sample solutions were prepared at a concentration of 10 mg/mL, while calibration solutions of gallic acid were prepared in distilled water at concentrations ranging from 12.5 to 400 μg/mL. The reaction mixture was prepared by combining the sample solutions with F-C reagent in a 1:5 volume ratio, followed by the addition of 100 μL of 75% Na_2_CO_3_. The mixture was incubated at room temperature for 30 min, and the absorbance was measured at 765 nm using a spectrophotometer (Infinite 200 PRO; TECAN, Männedorf, Switzerland). The TPC was calculated based on a calibration curve using gallic acid standards and expressed as milligrams of gallic acid equivalent per gram (mg GAE/g) of KP extract. The KP extract exhibited a TPC of 13.94 ± 0.33 mg GAE/g.

### 2.3. DPPH Radical-Scavenging Activity

The DPPH radical-scavenging activity of the KP extract powder was evaluated using a method adapted from Xiao et al. [15]. A 100-μL aliquot of a 0.20 mM DPPH radical solution in ethanol was combined with 10 mg/mL of KP extract powder. The mixture was incubated in the dark at room temperature for 30 min, after which the absorbance was measured at 517 nm using a spectrophotometer (Infinite 200 PRO; TECAN, Switzerland). Ascorbic acid served as the positive control, and all measurements were performed in triplicate. The concentration of extract required for 50% inhibition (IC_50_) was determined by plotting the inhibition percentages against various extract concentrations. The inhibition percentage was calculated using the following formula: Inhibition percentage (%) = (A_0_ − A_1_)/A_0_ × 100, where A_1_ represents the absorbance of the DPPH radical solution after incubation with the extract, and A_0_ represents the absorbance of the DPPH radical solution without the extract. The extract showed significant DPPH radical-scavenging activity, inhibiting 58.31% ± 2.87% of the DPPH radicals.

### 2.4. Animals

Six mature Thai native bulls (aged 3–4 years) were individually housed in pens (2.5 × 2.5 × 1.6 m) within an open-sided shed that received natural sunlight (approximately 12 h/day). Each animal was fed a commercial breeder ration at an average rate of 3 kg/animal/day, which is a concentrated feed containing 14% protein and 15 kg of rice straw. Additionally, the bulls had unlimited access to clean water. Semen samples were collected from these native Thai bulls at the Department of Animal Science, Faculty of Agriculture, Khon Kaen University, Thailand.

### 2.5. Semen Collection and Initial Quality Assessment

Semen samples were collected weekly via electroejaculation (ElectroJac IV, Neogen, Lexington, KY, USA) from each bull for six consecutive weeks, yielding a total of 36 ejaculates. Each ejaculate was immediately diluted 1:1 with a Tris egg yolk (TEY) extender. Samples were stored at 37 °C during transport to the laboratory, arriving within 15 min of collection. Fresh semen quality was evaluated in the laboratory, with sperm motility (total motility and progressive motility) assessed using phase-contrast microscopy at 400× magnification. Sperm viability was assessed using an eosin-nigrosin staining procedure [16]. Sperm concentrations were measured using a Neubauer-improved hemocytometer (Paul Marienfeld GmbH&Co. KG, Lauda-Königshofen, Germany). Samples were first diluted 1:200 in 4% sodium chloride solution to immobilize sperm before counting. The pH value was assessed using pH indicator paper. Six ejaculates that failed to meet the inclusion criteria (total motility and viability < 70%, concentration < 400 × 10^6^ sperm/mL) were excluded. Therefore, 30 ejaculates were included in the cryopreservation study.

The semen was collected during the rainy seasons between July and August. The average temperature–humidity index (THI) during this period was 81.79, and the average THI during the two months prior to semen collection was 80.19.

### 2.6. Experimental Design

This study aimed to determine the optimal *Kaempferia parviflora* (KP) concentration for improving post-thaw semen quality. The treatment doses were determined through a preliminary dose-response study, which allowed us to select a range of concentrations for investigation in the main experiment. The semen extender was supplemented with KP at different concentrations of 0 (control), 0.5, 1, and 1.5 mg/mL. Post-thaw semen quality was evaluated by assessing sperm motility, viability, membrane integrity, lipid peroxidation (MDA), and ROS production.

### 2.7. KP Preparation and Semen Extender Supplementation

The Tris-egg yolk (TEY) extender was prepared using 1.675 g citric acid monohydrate, 1.25 g fructose, 3.028 g tris, 0.10 g streptomycin, 0.06 g penicillin, and 20% (*v*/*v*) egg yolk per 100 mL ultrapure water. Two extender portions were prepared: one containing 14% glycerol and one without. Each portion was then supplemented with KP extract at 0, 0.5, 1, and 1.5 mg/mL concentrations.

To ensure complete dissolution of the KP in the semen extender, each mixture was vortexed at maximum speed (setting 10) for 60 s using a Lannet vortex mixture. Following vortexing, the mixture was allowed to stand undisturbed at room temperature for 30 min to ensure complete dissolution. The absence of visible KP precipitation was visually confirmed before overnight storage at 4 °C.

The pH and osmolarity of all extenders were determined immediately after preparation using a pH indicator paper and a vapor pressure osmometer (Wescor Inc., Logan, UT, USA). No statistically significant differences in pH (mean = 6.5) or osmolarity (mean = 315 mOsmol/kg) were observed among treatment groups (*p* > 0.05).

### 2.8. Semen Cryopreservation

The semen cryopreservation protocol followed the method described by Yangnam et al. [17]. Fresh semen samples meeting pre-freezing quality criteria were initially diluted with the TEY extender (without glycerol) at 37 °C to achieve a concentration of 80 × 10^6^ sperm/mL. The diluted semen was then cooled to 5 °C over 120 min. Following that, samples were subsequently mixed with an equal volume of the 14% glycerol-supplemented extender (1:1 *v*/*v*), resulting in a final glycerol concentration of 7%. All samples were equilibrated at 5 °C for 3 h. Subsequently, semen samples were loaded into 0.5-mL straws (IMV Technologies, L’Aigle, France) to achieve a final concentration of 20 × 10^6^ sperm per straw. The straws were then sealed with polyvinyl alcohol powder. The straws were cryopreserved via exposure to liquid nitrogen (LN_2_) vapor (4 cm above the LN_2_ surface) for 15 min before immersion in LN_2_ for storage. For analysis, straws were thawed in a 37 °C water bath for 30 s.

### 2.9. Sperm Motility

Post-thaw sperm quality was analyzed using computer-assisted sperm analysis (CASA; Olympus software version 10, HIM-IVOS; Hamilton Thorne Biosciences, Beverly, MA, USA). Total motility (MOT) and progressive motility (PMOT) were assessed. In total, 5 µL aliquots of diluted semen were placed on separate slides (maintained at 37 °C). Five microscopic fields per slide (more than 300 sperm) were captured for 10 s using an Olympus 10× phase-contrast lens and DP71/25 digital camera at 30 frames/s (fps; 60 Hz). Sperm with VAP < 5 µm/s were considered immotile, whereas those with VAP > 20 µm/s and STR ≥ 80% were classified as progressively motile.

### 2.10. Sperm Viability

Sperm viability was assessed using the eosin-nigrosin staining test [13]. The solution was prepared with Eosin Yellowish (Loba Chemie Pvt. Ltd., Mumbai, India) and Nigrosin Pure (Acros Organics, Geel, Belgium). For each sample, a 5 μL aliquot of semen—placed on a pre-warmed glass slide—was mixed with 10 μL of eosin-nigrosin stain (0.6% [*w*/*v*] eosin and 5% [*w*/*v*] nigrosin in distilled water) for 30 s. Subsequently, 15 μL of the mixture was smeared on another clean glass slide and dried at 37 °C. The dried smear was then observed for sperm viability using a bright-field microscope (400×). Viable sperms with intact plasma membranes remained unstained, whereas membrane-damaged sperms exhibited dark pink or red-stained sperm. A total of 300 sperm cells were counted per sample to determine the percentage of sperm viability.

### 2.11. Plasma Membrane Integrity

Plasma membrane integrity was assessed using the hypo-osmotic swelling test (HOST) [18]. The assay was conducted by mixing 50 µL of frozen-thawed semen with 0.5 mL of hypo-osmotic solution (150 mOsm/kg; 0.735 g sodium citrate dihydrate and 1.351 g fructose in 100 mL distilled water). After a 40-min incubation at 37 °C, sperm tail bending/coiling was noted by placing 15 µL of the mixture onto a warm slide (37 °C) and observing under light microscopy at 400× magnifications. Intact plasma membranes were indicated by coiled sperm tails. At least 200 sperm cells were observed per slide. Sperm with coiled tails after the HOST were considered to have intact plasma membranes.

### 2.12. Lipid Peroxidation

A total of 250 µL (approximately 10 × 10^6^ sperm) of semen was incubated with 0.25 mL of 0.2 mM ferrous sulfate (0906251; Ajex, Finechem Pty Ltd., Wollongong, Australia) and 0.25 mL of 1 mM ascorbic acid (Sigma, A5960) at 37 °C for 1 h. After incubation, 1 mL of 15% trichloroacetic acid (T6399; Sigma-Aldrich, St. Louis, MO, USA) and 1 mL of 0.375% TBA (T5500; Sigma-Aldrich, St. Louis, MO, USA) were added. The mixture was boiled at 100 °C for 10 min and then immediately cooled in water (4 °C). After centrifugation (4000× *g*, 4 °C, 10 min), MDA concentrations in the supernatant were determined using UV-visible spectrophotometry (Analytik Jena Specord 250 plus, Jena, Germany) at 532 nm.

### 2.13. ROS Production

Reactive oxygen species (ROS) production was measured using the Muse^®^ Oxidative Stress Kit (Luminex Corporation, Austin, TX, USA) according to the manufacturer’s instructions. This assay utilizes dihydroethidium to detect intracellular superoxide (O_2_^−^) in viable, adherent sperm cells (1–10 × 10^6^ cells/mL). After adding 190 μL of Muse^®^ Oxidative Stress Reagent, samples were homogenized, incubated for 30 min at 37 °C, and analyzed using a Muse^®^ Cell Analyzer. A threshold for ROS-positive (ROS+) cells was established by comparing the fluorescence intensity of untreated (ROS-negative) cells to cells treated with the reagent under ROS-inducing conditions (positive control). Cells exceeding this threshold were classified as ROS+ and below as ROS−.

### 2.14. Statistical Analysis

A completely randomized design was applied with four treatments: a control and three KP-treated groups (doses of 0.5, 1, and 1.5 mg/mL) in a semen diluent. Data normality was evaluated using the UNIVARIATE procedure and the Shapiro–Wilk test. Analysis of variance (ANOVA) was performed using SAS software V8.1 (SAS Institute, Inc., Cary, NC, USA) to analyze sperm total motility, progressive motility, viability, lipid peroxidation, and enzymatic activity. Tukey’s test (*p* < 0.05) was used for post-hoc comparisons of the mean values among treatments.

## 3. Results

### 3.1. Fresh Semen Quality

Fresh semen analysis revealed an average total motility of 79.58 ± 1.89%, progressive motility of 69.34 ± 2.00%, and viability of 77.51 ± 1.42%. Semen concentrations were recorded as 542.67 ± 9.62 million sperm/mL, with an average pH of 6.10 ± 0.10. These values served as the baseline for evaluating cryopreserved semen quality.

### 3.2. Frozen-Thaw Semen Quality

Post-thaw analysis demonstrated that the total motility, progressive motility, viability, and membrane integrity of the sperm cells were significantly affected by the addition of KP to the extender. The 1 mg/mL KP concentration yielded the best results across most parameters, with significantly higher total motility (46.29 ± 2.66%), progressive motility (43.34 ± 2.55%), viability (43.42 ± 2.15%), and membrane integrity (47.64 ± 1.18%) than those in the control group (*p* < 0.001). In contrast, the highest dose of KP (1.5 mg/mL) demonstrated a detrimental effect across most parameters, with decreased values for total motility (36.97 ± 3.32%), progressive motility (31.95 ± 2.31%), viability (30.88 ± 3.02%), and membrane integrity (35.64 ± 1.61%) relative to both the control and other treatment groups (Table 1).

**Figure 1:**
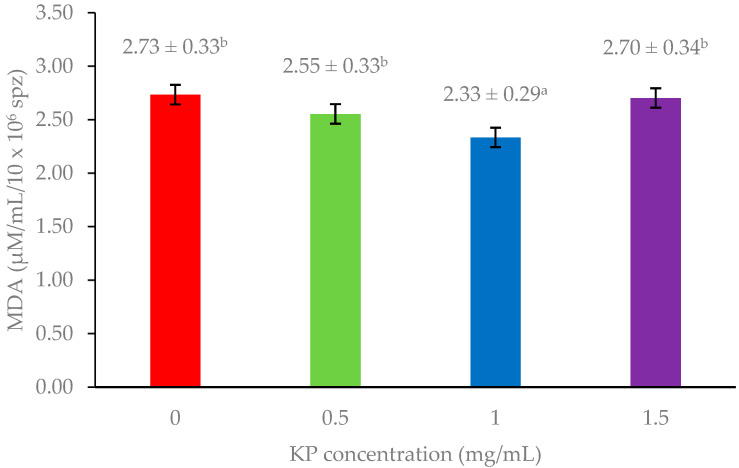
Effect of *Kaempferia parviflora* (KP) supplementation on malondialdehyde (MDA) levels in freeze-thawed bull sperm. This bar graph shows MDA levels (µM/mL/10 × 10^6^ sperm) in frozen-thawed bull sperm following treatment with varying concentrations of *Kaempferia parviflora* (KP) extract. Different lowercase letters above the bars indicate statistically significant differences (*p* < 0.05). Error bars represent standard error.

**Figure 2:**
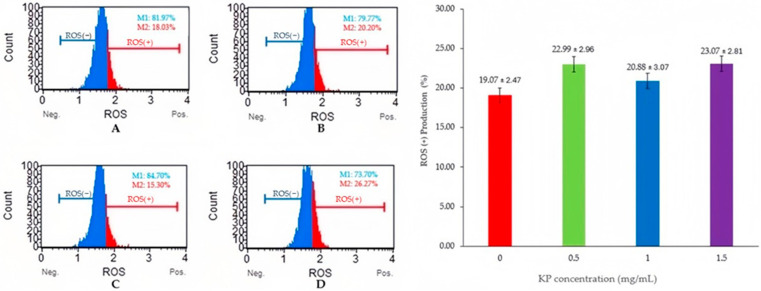
Flow cytometry analysis of semen samples with *Kaempferia parviflora* (KP) and ROS levels on frozen-thawed bull sperm. This figure presents the results of a flow cytometry analysis examining the effects of *Kaempferia parviflora* (KP) supplementation on reactive oxygen species (ROS) levels in frozen-thawed bull sperm. Four experimental groups are shown: (**A**) Control Group; (**B**) Supplementation with 0.5 mg/mL KP; (**C**) Supplementation with 1 mg/mL KP; (**D**) Supplementation with 1.5 mg/mL KP. The left panel displays four flow cytometry histograms (**A**–**D**), each showing the distribution of sperm cells according to their ROS levels. Within each histogram: ROS(−) represents the percentage of sperm cells with low ROS levels (M1); ROS(+) represents the percentage of sperm cells with high ROS levels (M2). The right panel presents a bar graph depicting the percentage of ROS(+) sperm cells across the four treatment groups. Error bars represent standard error.

Figure 1 and Figure 2 show the levels of MDA and ROS production which serve as indicators of lipid peroxidation and oxidative stress, respectively. Adding 1 mg/mL KP resulted in significantly lower MDA levels compared with other treatments (*p* < 0.05), whereas ROS production showed non-significant differences among the groups (*p* > 0.05).

## 4. Discussion

This study demonstrates that supplementing semen extenders with KP significantly improves the post-thaw quality of native bull sperm. Specifically, adding 1 mg/mL KP improved sperm motility, viability, and membrane integrity compared to the control and other treatment groups. These findings suggest that the antioxidant properties of KP effectively mitigate oxidative stress and cryodamage typically associated with semen cryopreservation. Consistent with findings of different studies concerning other livestock species, natural antioxidants like KP reduce the production of ROS, thereby protecting sperm membranes from lipid peroxidation and preserving sperm function [8]. The significant reduction in oxidative stress markers, including MDA and ROS, observed with the addition of 1 mg/mL KP further supports the hypothesis that certain KP concentrations can enhance cryoprotection. However, at higher concentrations (1.5 mg/mL), KP appeared to exert a detrimental effect—likely due to the excessive antioxidant activity or pro-oxidant effects at higher doses—highlighting the importance of precise dosing in cryopreservation protocols involving natural supplements.

A key factor contributing to sperm plasma membrane damage following cryopreservation is the imbalance between antioxidants and free radicals during the freezing process, which induces oxidative stress and lipid peroxidation [19]. The sperm plasma membrane is rich in polyunsaturated fatty acids, making it highly vulnerable to reactive oxygen species (ROS), which negatively impact motility, morphology, membrane integrity, and overall fertility [20]. Normally, seminal plasma contains a range of antioxidants, including superoxide dismutase, catalase, and glutathione peroxidase, which help neutralize ROS [21]. However, these antioxidants are significantly depleted after semen collection, while excessive ROS may be produced by dying sperm exposed to stressors such as dilution, cooling, and prolonged incubation during freezing [22]. Therefore, incorporating antioxidants into cryopreservation extenders is essential to mitigate oxidative damage and preserve sperm quality. In this study, adding 1 mg/mL of KP significantly improved post-thaw sperm quality parameters compared with control and other treatments. This improvement can be attributed to KP’s antioxidant properties. KP contains bioactive flavonoids such as methoxyflavones [23], which neutralize excessive ROS produced during freezing and thawing. It directly scavenges ROS, such as superoxide and hydroxyl radicals, thus preventing lipid peroxidation and protein denaturation [24,25]. Moreover, it potentially upregulates endogenous antioxidant enzyme activity in sperm cells [24]. The significant reduction in MDA levels with 1 mg/mL KP (Figure 1) indicated a decrease in lipid peroxidation in sperm cells, consistent with the improved sperm quality parameters (motility, viability, and membrane integrity) observed at this concentration (Table 1). These findings support the hypothesis that KP’s antioxidant activity protects sperm membranes from oxidative damage.

The antioxidant properties of KP are further corroborated by its ability to scavenge free radicals, as indicated by the low IC_50_ values in DPPH and ABTS radical scavenging assays [7]. The KP that was used in the present study had a high total phenolic content of 13.94 mg GAE/g and substantial DPPH radical scavenging activity of 58.31% in KP extracts. This substantial antioxidant capacity, linked to KP’s high phenolic content, likely directly protects sperm from oxidative damage during cryopreservation.

In contrast to the beneficial effects observed at 1 mg/mL KP, a KP concentration of 1.5 mg/mL resulted in a significant decline across all sperm quality parameters compared to both the control and optimal 1 mg/mL KP concentration. The absence of significant differences in MDA levels between the 1.5 mg/mL KP and control groups, despite the decline in sperm quality parameters, warrants further investigation. Several factors could account for this. First, the antioxidant effect may reach saturation at higher KP concentrations, a phenomenon documented in antioxidant research [26,27], in which increased antioxidant concentration beyond a certain threshold does not further inhibit lipid peroxidation. KP may have reached this saturation at 1.5 mg/mL, resulting in MDA levels similar to those of the control. Second, the pro-oxidant effects triggered at high concentrations cannot be ruled out; some antioxidants become pro-oxidant at high concentrations, potentially promoting oxidative stress rather than mitigating it [28,29]. This biphasic response may explain the comparable MDA levels in the 1.5 mg/mL KP and control groups. In this scenario, elevated KP concentrations may fail to counteract lipid peroxidation, and instead, exacerbate oxidative stress. These findings are consistent with previous research demonstrating that excessive antioxidant supplementation can be detrimental to sperm function in bulls. For example, high doses of ascorbic acid have been associated with frozen sperm damage [29,30], while elevated concentrations of sericin [17,31] and DHA [32] have also been shown to impact sperm parameters negatively. These observations underscore the dose-dependent nature of antioxidant efficacy and the critical need to identify optimal concentrations for maximal protective effects.

While both MDA and ROS levels are indicators of oxidative stress, the lack of correlation between MDA and ROS production warrants further discussion. MDA specifically reflects lipid peroxidation, whereas ROS encompasses a broader range of reactive oxygen species. This is consistent with prior research on the distinct roles of MDA and ROS in oxidative stress [28]. The significant reduction in MDA levels at 1 mg/mL KP reflects effective mitigation of lipid peroxidation. However, the lack of discernible ROS production levels compared to the other treatment groups may indicate either a rise in another specific ROS species that does not contribute to lipid peroxidation or an alteration in the redox balance within cells at 1 mg/mL KP. This illustrates the intricate interplay of various reactive species involved in the oxidative stress response.

Although this study demonstrates the beneficial effects of *Kaempferia parviflora* supplementation in improving post-thaw sperm quality, certain limitations should be acknowledged. One key limitation is the lack of mechanistic insights into how KP exerts its protective effects on sperm during cryopreservation. While the results indicate that KP supplementation enhances motility, viability, and membrane integrity, the specific molecular pathways involved remain unclear. Antioxidants, particularly flavonoids present in KP, are known to reduce oxidative stress by scavenging reactive oxygen species (ROS) and protecting cellular structures from lipid peroxidation [33]. However, further investigations using molecular and biochemical analyses, such as oxidative stress markers, mitochondrial function assays, and gene expression studies, are necessary to elucidate the precise mechanisms underlying KP’s protective role [34]. Without such insights, the full potential of KP in sperm cryopreservation cannot be fully harnessed, and its application may remain limited to empirical observations.

Another important limitation of this study is the lack of fertility assessment following cryopreservation. While improvements in sperm motility, viability, and membrane integrity are promising indicators of fertilizing potential, they do not necessarily translate into enhanced fertility outcomes [35,36]. In vivo fertility trials, such as artificial insemination studies or in vitro fertilization experiments, are essential to determine whether KP supplementation improves actual fertilization rates, embryo development, and pregnancy success. Therefore, future studies should incorporate fertility trials to validate the practical benefits of KP supplementation in livestock breeding programs. Integrating mechanistic studies and fertility assessments will provide a more comprehensive understanding of KP’s role in cryopreservation, enabling its optimized application in reproductive technologies.

## 5. Conclusions

Semen extender supplementation with 1 mg/mL of KP significantly improves post-thaw semen quality in Thai native bulls, likely due to its antioxidant properties. However, higher concentrations (1.5 mg/mL) had adverse effects, suggesting that excessive KP supplementation may lead to oxidative imbalances or toxicity. These findings highlight the critical role of optimal antioxidant dosing in cryopreservation protocols to maximize sperm quality while minimizing potential damage. Future studies should explore its effects on fertility outcomes in artificial insemination and in vitro fertilization and the underlying molecular mechanisms using OMICS technologies. Understanding these aspects will further optimize cryopreservation strategies, ultimately benefiting livestock breeding programs and genetic conservation efforts.

## Figures and Tables

**Table 1:** Effect of KP concentration on post-thaw semen quality in Thai native bulls (mean ± SE).

KP Concentration (mg/mL)	Total Motility (%)	Progressive Motility (%)	Viability (%)	Membrane Integrity (%)
0	40.77 ± 2.76 ^b^	38.49 ± 2.63 ^b^	38.63 ± 2.66 ^b^	41.85 ± 1.98 ^b^
0.5	40.92 ± 2.53 ^b^	39.31 ± 2.44 ^b^	39.65 ± 2.35 ^ab^	43.42 ± 1.81 ^b^
1	46.29 ± 2.66 ^a^	43.34 ± 2.55 ^a^	43.42 ± 2.15 ^a^	47.64 ± 1.18 ^a^
1.5	36.97 ± 3.32 ^b^	31.95 ± 2.31 ^c^	30.88 ± 3.02 ^c^	35.64 ± 1.61 ^c^
*p*-Value	<0.001	<0.001	<0.001	<0.001

Means with different superscript letters differ significantly (*p* < 0.05; ANOVA followed by Tukey’s HSD post-hoc test).

## Data Availability

The data are available upon request from the corresponding author.

## Abbreviations

The following abbreviations are used in this manuscript:

KP	*Kaempferia parviflora*
TEY	Tris-egg yolk extender
MOT	Total motility
PMOT	Progressive motility
VAP	Average path velocity
STR	Straightness
ROS	Reactive oxygen species
MDA (µM/mL)	Malondialdehyde (micromoles per milliliter)
TPC (mg GAE/g)	Total phenolic content (milligrams gallic acid equivalent per gram)
DPPH	2,2-Diphenyl-1-picrylhydrazyl
LN_2_	Liquid nitrogen
